# Efficacy and Safety of Gastrointestinal Tumour Site Marking with da Vinci-Compatible Near-Infrared Fluorescent Clips: A Case Series

**DOI:** 10.1007/s00268-023-07082-6

**Published:** 2023-06-20

**Authors:** Junji Takahashi, Masashi Yoshida, Hironori Ohdaira, Yuichi Nakaseko, Keigo Nakashima, Teppei Kamada, Norihiko Suzuki, Takayuki Sato, Yutaka Suzuki

**Affiliations:** 1 Department of Surgery, International University of Health and Welfare Hospital, 537-3, Iguchi, Nasushiobara City, Tochigi 329-2763 Japan; 2 Center for Photodynamic Medicine, Kochi University, Kohasu Oko-Cho 185-1, Nankoku, Kochi 783-8505 Japan

## Abstract

**Background:**

The conventional near-infrared fluorescent clip (NIRFC) ZEOCLIP FS® has been used successfully in marking tumour sites during laparoscopic surgeries. However, this clip is difficult to observe with the Firefly imaging system equipped with the da Vinci® surgical system. We have been involved in the modification of ZEOCLIP FS® and development of da Vinci-compatible NIRFC. This is the first prospective single-centre case series study verifying the usefulness and safety of the da Vinci-compatible NIRFC.

**Methods:**

Twenty-eight consecutive patients undergoing da Vinci®-assisted surgery for gastrointestinal cancer (16 gastric, 4 oesophageal, and 8 rectal cases) between May 2021 and May 2022 were enrolled.

**Results:**

Tumour location was identified by the da Vinci-compatible NIRFCs in 21 of 28 (75%) patients, which involved 12 gastric (75%), 4 oesophageal (100%), and 5 rectal (62%) cancer cases. No adverse events were observed.

**Conclusion:**

Tumour site marking with da Vinci-compatible NIRFC was feasible in 28 patients enrolled in this study. Further studies are warranted to substantiate the safety and improve the recognition rate.

## Introduction

**Fig. 1:**
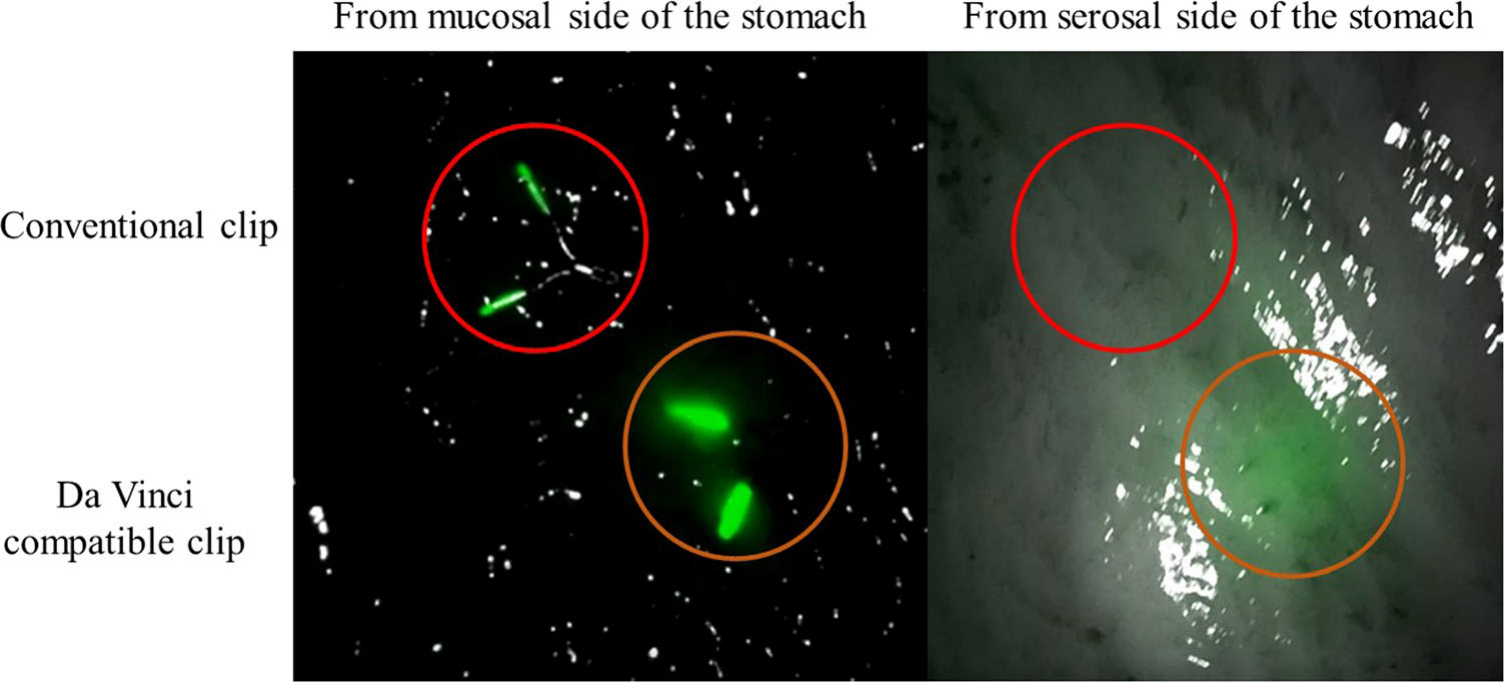
Ex vivo bench observations were performed in the resected stomach. From the serosal side of the stomach, a conventional NIRFC (red circle) cannot be seen with the Firefly imaging system, whereas a da Vinci-compatible NIRFC can be seen (orange circle)

**Fig. 2:**
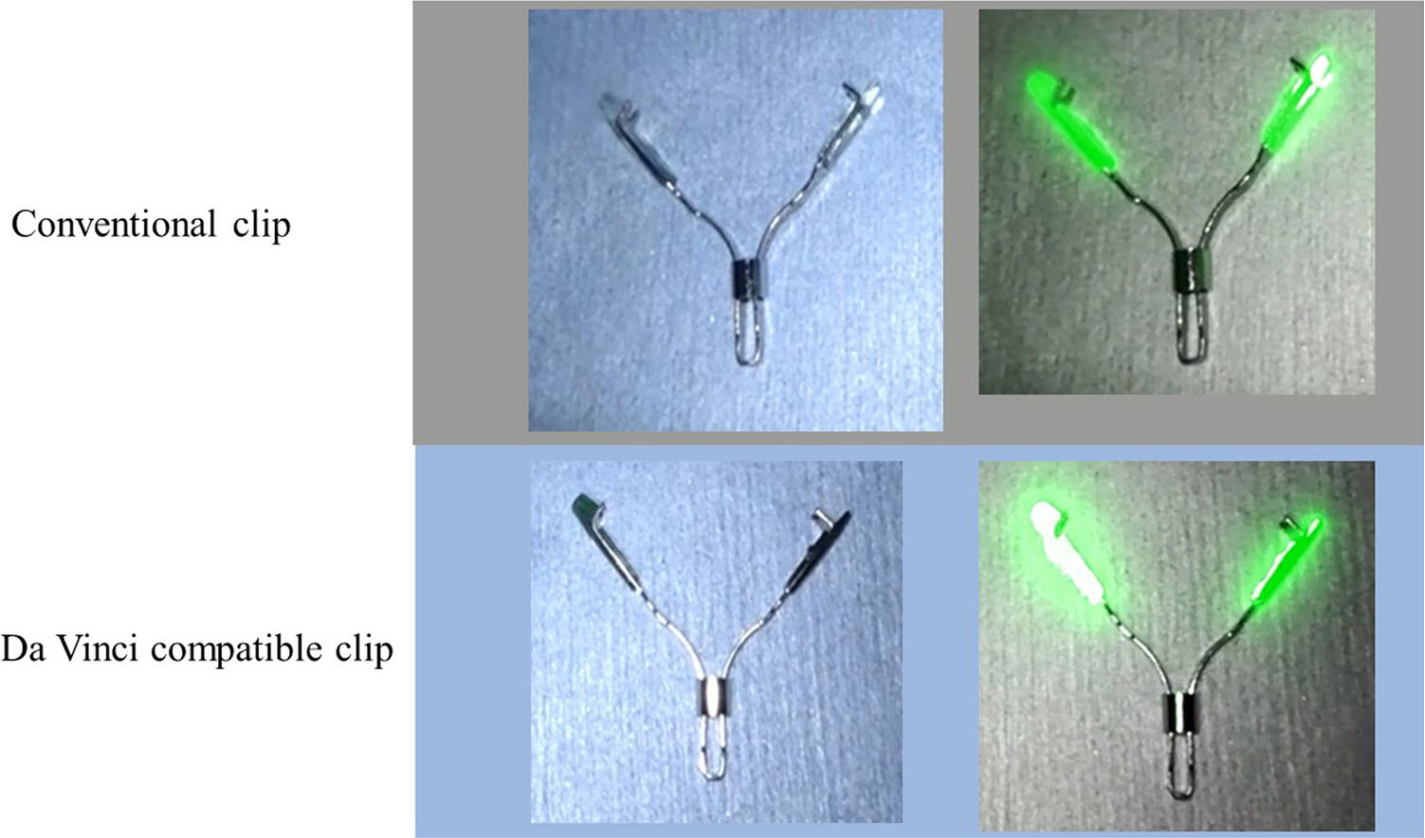
A da Vinci-compatible NIRFC was developed. A near-infrared fluorescent resin appears darker green (increased intensity) than that of a conventional clip due to the increased fluorescent dye (boron-dipyrromethene) concentration in the da Vinci-compatible NIRFC

Information on tumour location during robot-assisted surgery is crucial for surgical procedures. Anayama et al., have developed near-infrared fluorescent resins that can be used for clips and catheters [1]. We have been involved in the development of the near-infrared fluorescent clip (NIRFC) ZEOCLIP FS® (Zeon Medical, Tokyo, Japan) for the recognition of the location of gastrointestinal tumour intraoperatively. Although the NIRFC is effective in laparoscopic surgery [2, 3], this clip was difficult to observe in patients during laparoscopic surgery using the da Vinci Firefly® imaging system (Intuitive Surgical, California, USA) [4] (Fig. 1). One of the reasons for this may be the differences in the peak excitation and emission wavelengths between the Firefly system and fluorescent clip. The Firefly system is equipped with a laser beam (excitation wavelength of 805 nm) and is designed to observe indocyanine green fluorescence with a peak at 830 nm. This excitation wavelength may differ from the ideal excitation wavelength of conventional NIRFC, which has a peak excitation wavelength of 760 nm and a peak fluorescence of 790 nm [3]. Different concentrations of a dye (boron-dipyrromethene) were examined, and this problem was addressed by increasing the fluorescent dye concentration (Fig. 2). Previously, we reported a case in which submucosal tumour in the stomach was localised using the da Vinci-compatible NIRFCs, facilitating local resection [4]. In this study, we examined 28 cases of tumour site marking with the da Vinci-compatible NIRFCs and examined the efficacy and safety of this method. The primary aim was to determine whether the tumour site could be identified intraoperatively using clip detection. The secondary aim was the analysis of clip detection rate, clip slippage, and adverse effect incidence. To our knowledge, this is the first report of a case series, in which the da Vinci-compatible NIRFCs were used for gastrointestinal tumour site marking.

## Methods

### Study design

This was a prospective single-centre case series study. Between May 2021 and May 2022, all patients undergoing da Vinci-assisted surgery for the gastrointestinal tract were enrolled without any exclusion criteria. The primary endpoint was the clip recognition rate. Secondary endpoints were complications, clip dislodgement rate, and correlation between the number of days between clip placement, surgery, and recognition rate. The study protocol was approved by the institutional ethics committee (No. 13-B-344). Trial registration number: UMIN-CTR study design; trial number: UMIN000048531.

Written informed consent for participation in the study was obtained from all the patients. All patients were treated and followed up at a single hospital in a university institution. For gastric cancer, the sample was stratified into two groups, namely, the recognition group in whom NIRFC was recognised and the non-recognition group in whom NIRFC was not recognised. The distance between the proximal edge of the tumour to the dissection line (dissection margin) in the resected specimens was compared.

### Endoscopic clip placement technique

Endoscopic clip placement was performed using upper or lower gastrointestinal endoscopy by surgeons having at least 5 years of experience within the same institution.

The da Vinci procedure was performed by the same surgeon (H.O.), a senior gastrointestinal surgeon with more than 280 da Vinci surgery experience. The date of endoscopic clip placement was 1–3 days before surgery in 27 cases and 6 days before surgery in one case due to scheduling reasons.

**Fig. 3:**
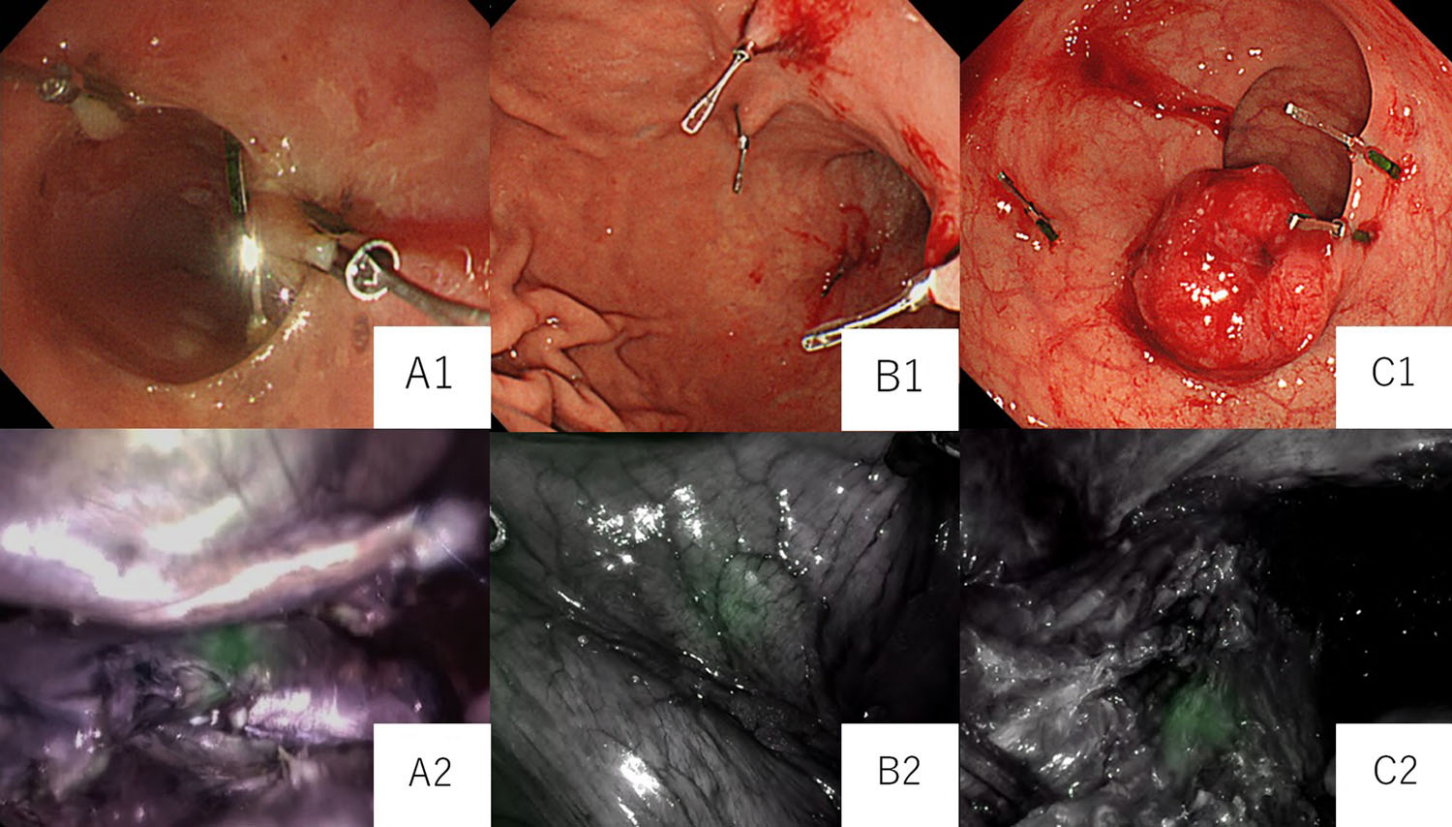
A1: An endoscopic oesophageal image. Clips are placed proximal and distal to the tumour. A2: A Firefly image during thoracic manipulation of oesophageal surgery. The clips in the distal part of the oesophageal tumour fluoresce green. B1: A gastroendoscopic image. Clips are placed in the proximal part of the tumour. B2: A Firefly image during gastric surgery manipulation. The clips in the proximal part of the gastric tumour fluoresce green. C1: A colonoscopy image. Clips are placed distal to tumour. C2: A Firefly image during rectal surgery abdominal manipulation. The clips in the distal part of the tumour in the rectum fluoresce green

For oesophageal tumours, clips were placed on the mucosa on the proximal and distal sides of the tumour, on the ventral and posterior sides and on the right side (Fig. 3a). For gastric tumours, clips were placed on the proximal side of the tumour in all cases. For a case with submucosal tumour, 4 clips were located around the tumour. (Fig. 3b). In the case of local resection, clips were placed on the proximal and distal sides of the tumour. For rectal tumours, four clips were placed on the distal side at 90° around the tumour (Fig. 3c).

### Clip detection

For intraoperative clip detection, the clips were identified by observation with Firefly mode on. In the oesophageal cancer cases, tumour sites were observed with the fluorescence camera at the time of camera insertion, while in the gastric and rectal cancer cases, they were observed after dissection of the surrounding tissue to increase tissue mobility for observation.

After the tumour site was detected by a translucent signal emitted from the fluorescent clip, the tumour was resected, including the relevant area. In all cases, the position and number of clips were checked in the resected surgical specimens to count slipped clips.

When the fluorescent clip could not be recognised during the operation, the line of dissection was determined by intraoperative endoscopy for rectal cancer. In the case of gastric cancer, the surgeon postulated the dissection line from the preoperative images and dissected the tumour. The size of the remnant stomach was intended to be large when the dissection line was determined.

### Statistical analysis

The correlation between the number of days between clip placement, surgery, and recognition rate was examined in all cases with the Mann–Whitney *U* test. The dissection margin between the two groups was expressed as a mean and its standard deviation (± SD). The difference between the two groups was analysed with a one-sided *t*-test (the size of the remnant stomach was intended to be large when the dissection line was determined; therefore, the dissection margin in the recognition group cannot be longer than that in the non-recognition group). A significance level of 5% was applied. Statistical analyses were performed using the statistical package STATA/IC 14.2 (StataCorp LLC, Texas, USA).

## Results

Twenty-eight (22 males and 6 females) patients were enrolled in the present study. No patient objected to the study after it was explained to them. The median age was 70 years (range, 43–82 years). Tumour location was assessed in 4 oesophagus, 16 gastric (2 upper gastric, 11 middle gastric, and 3 lower gastric cancer cases), and 8 rectal (6 Rs, 1 Ra, and 1 Rb cancer cases) cancer cases. The patient characteristics are detailed in Table 1.

**Table 1:** Demographics of study patients

Parameter		
Sex (Male:Female)		22:6
Median age (range), years		70 (43–82)
BMI (range)		22.3(16.0–30.2)
Comorbidities (number of cases)	Abdominal surgical history	7
	Atrial fibrillation	1
	Chronic obstructive pulmonary disease	1
	Hypertension	12
	Diabetes mellitus	7
	Dyslipidaemia	8
Tumour lesion	Oesophageal tumour (Te:Ae)	3:1
	Gastric tumour (U:M: L)	2:11:3
	Rectal Tumour (Rs:Ra:Rb)	6:1:1
Tumour classification (UICC)	Oesophageal cancer (T1a:1b:3)	2:1:1
	Gastric cancer (T1a:1b:2:3:4a)	2:11:3
	Rectal cancer (T1b:2:3)	3:1:2
Cancer stage (UICC)	Oesophageal cancer(I:II:III:IV)	3:1:0:0
	Gastric cancer((I:II:III:IV)	11:2:0:0
	Rectal cancer((I:II:III:IV)	4:1:0:3
Preoperative endoscopy (day1:2:3:6)		7:16:4:1

Preoperative endoscopy was performed the day before surgery in 7 cases, 2 days before surgery in 16 cases, 3 days before surgery in 4 cases, and 6 days before surgery in 1 case.

There was no bleeding, perforation, or other complications during the clip placement. The tumour location was identified by fluorescence in 21 of 28 (75%) patients: 4 oesophageal (100%), 12 gastric (75%), and 5 rectal (62%) cancer cases (Fig. 3). Clips were not dislodged in all cases (*n* = 93/93). No adverse effect due to clips were observed.

For the number of days from clip placement to surgery and recognition rate, the recognition group averaged 1.76 days (± 0.62) and the non-recognition group 2.57 days (± 1.61) (*p* = 0.17). For distal margins and recognition rates, there were 14 cases of gastric cancer, 10 in the recognition group and 4 in the non-recognition group. The mean distal margin of the recognition group was 4.67 cm (± 2.31), while that of the non-recognition group was 7.40 cm (± 2.57) (*p* = 0.03). In the recognition group, smaller resection could be performed, and larger remnant stomach could be reserved.

## Discussion

In this case series, tumour site marking with the NIRFC was feasible and safe in da Vinci surgery for gastrointestinal tumours.

One method of marking tumour location is endoscopic tattooing, which has a spillage to the abdominal cavity at a rate of 2.4–13% [5]. Complications of endoscopic tattooing include focal peritonitis [6, 7], infected haematoma and abscess formation [7–9], inflammatory pseudotumor [10], idiopathic inflammatory enteritis [11], postoperative adhesion [12], tumour inoculation [13], and small intestine perforation [6].

Based on the reported eight articles [3, 4, 14–19], tumour site marking was performed with conventional NIRFC without any adverse effects, which is in line with the present study.

Tumour site marking by da Vinci compatible NIRFC was possible in majority of the cases (75%). There could have been potential to improve the recognition rate. For example, the soft tissue penetration distance of near-infrared light is 5–10 mm [20], and fluorescence cannot be seen if the gastrointestinal wall is thick. In addition, care should be taken with the angle between the camera and intestine. If the intestinal wall thickness is 5 mm, the angle at which near-infrared light can be observed within a biological transmission distance of 10 mm must be at least 30° [14]. For these conditions, Namikawa et al. [1] sent 100 ml air into the stomach through a nasogastric tube during observation, making the stomach wall extended and thin. Hara et al. [21] found the following ingenuity, that improved the viewability of a fluorescent clip. First, the gastric wall should be stretched and the camera positioned as far as possible in front of the clip. Second, the camera should be angled vertically and upright by pushing the stomach from behind with forceps to make the wall thin. In addition, pressing the gastric wall with the forceps, where slight fluorescence was seen, and opening the forceps are useful manoeuvres for fluorescence confirmation (Hara's manoeuvre). A fluorescent clip on the anterior wall is the easiest location to view, but the posterior wall clips can also be definitely seen by turning the stomach inverted and irradiating the near-infrared light at an appropriate angle. Moreover, for tumours located in the lesser and greater curvatures, which are difficult to observe due to the fatty tissue, it is necessary to displace the clips slightly to the anterior or posterior wall side or to detach the fatty tissue. Using these techniques, they reported that NIRFCs could visualise all 32 gastric cancer cases.

In the rectum, Narihiro et al. [3] applied excitation light vertically as much as possible, increasing the recognition rate. Their device may further increase the recognition rate. In the present study, of the eight cases where the location could not be ascertained, four were owing to the tangentially oriented camera, and four were in the gastric antrum with thick surrounding tissue or the rectum with high mesorectal fat. In this study, air was not pumped into the stomach, as in Namikawa et al. [1], and Hara’s manoeuvres were not performed [21]. Therefore, the present results reflect the outcome of simple observations, and the recognition rate may be improved using Hara’s maneuvers.

As for the number of days from clip placement to surgery and recognition rate, no significant correlation was observed. This factor may also not influence the detection rate. In the present study, the tumour was visible from the serosal side in one gastric cancer case (Table 1: T4a). As the green fluorescence of NIRFC was clearly visible in this case, it is assumed that the tumour invasion depth did not influence the NIRFC recognition rate.

In this study, the dissection margin was significantly shortened in gastric cancer cases where the clip could be recognised. This may have contributed to the remnant stomach being large. Large gastric remnants may reduce diarrhoea and improve meal-related scores on the Postgastrectomy Syndrome Assessment Scale-45 [22]. With further manoeuvres in recognition, using clips may reduce excessive resection compared to that in the case of surgeon's postulated dissection.

## Conclusion

Using da Vinci-compatible NIRFCs, tumour location was identified in 75% patients without any adverse effects. The identification rate can be improved more by a little ingenuity.

## Abbreviation

NIRFC: Near-infrared fluorescent clip

## Declarations

### Provenance and peer review

Not commissioned, externally peer-reviewed

## References

[1] Namikawa T, Iwabu J, Hashiba M (2020). Novel endoscopic marking clip equipped with resin-conjugated fluorescent indocyanine green during laparoscopic surgery for gastrointestinal cancer. Langenbecks Arch Surg.

[2] Furbetta N, Palmeri M, Morelli L (2018). Gastrointestinal stromal tumours of the stomach: Robot-assisted excision with the da Vinci Surgical System regardless of size and location site. J Minim Access Surg.

[3] Narihiro S, Yoshida M, Ohdaira H (2020). Effectiveness and safety of tumour site marking with near-infrared fluorescent clips in colorectal laparoscopic surgery: a case series study. Int J Surg.

[4] Takahashi J, Yoshida M, Nakaseko Y (2022). Near-infrared fluorescence clip-guided robot-assisted wedge resection of a gastric submucosal tumour: a case report. Int J Surg Case Rep.

[5] Trakarnsanga A, Akaraviputh T (2011). Endoscopic tattooing of colorectal lesions: s it a risk-free procedure?. World J Gastrointest Endosc.

[6] Singh S, Arif A, Fox C et al (2006) Complication after pre-operative India ink tattooing in a colonic lesion. Dig Surg 23:303 (Abstract). 10.1159/000096245. Google Scholar. 10.1159/000096245 17047331

[7] Park SI, Genta RS, Romeo DP et al (1991) Colonic abscess and focal peritonitis secondary to India ink tattooing of the colon. Gastrointest Endosc 37:68–71 (Abstract). 10.1016/s0016-5107(91)70627-5. Google Scholar. 10.1016/s0016-5107(91)70627-5 1706286

[8] Marques I, Lagos AC, Pinto A et al (2011) Rectal intramural haematoma: a rare complication of endoscopic tattooing, Gastrointest Endosc 73:366–367 (Abstract). 10.1016/j.gie.2010.07.027 (Google Scholar). 10.1016/j.gie.2010.07.027 21126738

[9] Alba LM, Pandya PK, Clarkston WK (2000) Rectus muscle abscess associated with endoscopic tattooing of the colon with India ink. Gastrointest Endosc 52:557–558 (Abstract). 10.1067/mge.2000.108660 (Google Scholar). 10.1067/mge.2000.108660 11023584

[10] Coman E, Brandt LJ, Brenner S et al (1991) Fat necrosis and inflammatory pseudotumour due to endoscopic tattooing of the colon with India ink. Gastrointest Endosc 37 65–68 (Abstract). 10.1016/s0016-5107(91)70626-3 (Google Scholar). 10.1016/s0016-5107(91)70626-3 1706285

[11] Gopal DV, Morava-Protzner I, Miller HA et al (1999) Idiopathic inflammatory bowel disease associated with colonic tattooing with India ink preparation—case report and review of literature. Gastrointest Endosc 49:636–639 (Abstract). 10.1016/s0016-5107(99)70395-0 (Google Scholar). 10.1016/s0016-5107(99)70395-0 10228265

[12] Yano H, Okada K, Monden T (2003) Adhesion ileus caused by tattoo-marking: unusual complication after laparoscopic surgery for early colorectal cancer. Dis Colon Rectum 46:987 (Abstract). 10.1007/s10350-004-6699-6 (Google Scholar). 10.1007/s10350-004-6699-6 12847379

[13] Tutticci N, Cameron D, Croese J et al (2010) Peritoneal deposits with carbon pigmentation associated with endoscopic submucosal tattooing of a rectal cancer, Endoscopy 42(Suppl 2):E136. Abstract. 10.1055/s-0029-1244049 (Google Scholar). 10.1055/s-0029-1244049 20405382

[14] Ryu S, Okamoto A, Nakashima K (2020). Usefulness of preoperative endoscopic fluorescent clip marking in laparoscopic gastrointestinal surgery. Anticancer Res.

[15] Shinozaki S, Sunada K, Otake T (2015). Utility of a new reusable clip device for endoscopic treatment. Dig Endosc.

[16] Narihiro S, Yoshida M, Ohdaira H (2020). Near-infrared fluorescent clip-guided gastrectomy: Report of 2 cases (case reports). Ann Med Surg (Lond).

[17] Shunjin Ryu K, Ishida AO (2020). Laparoscopic fluorescence navigation for left-sided colon and rectal cancer: blood flow evaluation, vessel and ureteral navigation, clip marking and trans-anal tube insertion. Surg Oncol.

[18] Hamada S, Ihara E, Yoshitake C et al. (2022) Clip stopper closure method using a detachable snare in combination with ZEOCLIP for endoscopic submucosal dissection-induced mucosal defects. Dig Endosc. 10.1111/den.14417 [Online ahead of print]. 10.1111/den.14417 36039010

[19] Narihiro S, Yoshida M, Ohdaira H (2019). A novel fluorescent marking clip for laparoscopic surgery of colorectal cancer: a case report. Int J Surg Case Rep.

[20] Kim S, Lim YT, Soltesz EG (2004). Near-infrared fluorescent type II quantum dots for sentinel lymph node mapping. Nat Biotechnol.

[21] Hara K, Shunjin R, Okamoto A (2022). Intraoperative tumour identification during laparoscopic distal gastrectomy: a novel fluorescent clip marking versus metal clip marking and intraoperative gastroscope. J Gastrointest Surg.

[22] Misawa K, Terashima MY, Uenosono Y (2015). Evaluation of postgastrectomy symptoms after distal gastrectomy with Billroth-I reconstruction using the Postgastrectomy Syndrome Assessment Scale-45 (PGSAS-45). Gastric Cancer.

