# Correction: Efficacy and Safety of Gastrointestinal Tumour Site Marking with da Vinci-Compatible Near-Infrared Fluorescent Clips: A Case Series

**DOI:** 10.1007/s00268-023-07184-1

**Published:** 2023-09-27

**Authors:** Junji Takahashi, Masashi Yoshida, Hironori Ohdaira, Yuichi Nakaseko, Keigo Nakashima, Teppei Kamada, Norihiko Suzuki, Takayuki Sato, Yutaka Suzuki

**Affiliations:** 1 Department of Surgery, International University of Health and Welfare Hospital, 537-3, Iguchi, Nasushiobara City, Tochigi 329-2763 Japan; 2 Center for Photodynamic Medicine, Kochi University, Kohasu Oko-Cho 185-1, Nankoku, Kochi 783-8505 Japan

**Correction to: World J Surg (2023) 47:2386–2391** 10.1007/s00268-023-07082-6

In the original online version of this article the authors' family names and affiliations were incorrect. The original article was corrected.

